# Joint associations of estimated glucose disposal rate and high-sensitivity C-reactive protein with metabolic syndrome in Korean adults: A cross-sectional study

**DOI:** 10.1097/MD.0000000000051429

**Published:** 2026-11-06

**Authors:** Linling Ruan, Shuangyu Ou

**Affiliations:** a Department of Clinical Laboratory, Ningde Municipal Hospital, Ningde, Fujian, China.

**Keywords:** cross-sectional study, estimated glucose disposal rate (eGDR), high-sensitivity C-reactive protein (hs-CRP), insulin resistance, metabolic syndrome

## Abstract

Metabolic syndrome (MetS), an important metabolic risk phenotype within the broader cardiovascular-kidney-metabolic (CKM) framework, is characterized by insulin resistance and chronic low-grade inflammation; however, evidence regarding the joint relationship of these 2 biological dimensions with MetS remains limited. We analyzed cross-sectional data from 20,781 Korean adults aged ≥ 19 years who were enrolled in the Korea National Health and Nutrition Examination Survey (KNHANES) 2015–2018. MetS was classified using the National Cholesterol Education Program Adult Treatment Panel III framework with waist circumference thresholds adapted for the Korean/Asian populations. Weighted logistic regression was performed to examine the relationships between estimated glucose disposal rate (eGDR) and high-sensitivity C-reactive protein (hs-CRP) and MetS. Potential dose–response patterns were further explored using restricted cubic splines. Joint analyses, interaction analysis, and prespecified subgroup analyses were also performed. MetS was present in 29.4% of participants after weighting. In the fully adjusted model, higher eGDR was associated with lower odds of MetS per unit (OR 0.48, 95% CI: 0.47–0.49), whereas log-transformed hs-CRP showed a positive association with MetS, with higher levels corresponding to greater odds (OR 1.71, 95% CI: 1.64–1.79; both *P* < .001). Dose–response analyses revealed a decreasing pattern for eGDR and an increasing pattern for hs-CRP in relation to MetS. In joint analyses, progressively higher odds of MetS were observed across exposure categories, with the low-eGDR/high-hs-CRP profile corresponding to the greatest odds of MetS. No significant multiplicative interaction was detected (*P* for interaction = .064). The association of the joint high-risk profile with MetS was generally consistent across subgroups and appeared stronger among younger participants and those without diabetes. Lower eGDR and higher hs-CRP levels were independently associated with MetS in Korean adults, and the low-eGDR/high-hs-CRP profile was associated with the highest odds of MetS. Their joint assessment may provide complementary information for characterizing adverse metabolic profiles within the broader CKM framework, although prospective studies are needed to determine its predictive value.

## 1. Introduction

Metabolic syndrome (MetS) refers to the co-occurrence of central adiposity, elevated blood pressure, dysglycemia, and lipid abnormalities and represents a major global public health challenge with a growing burden worldwide.^[1]^ A substantial body of evidence indicates that MetS is associated with a higher likelihood of incident type 2 diabetes, cardiovascular events, cardiovascular mortality, and all-cause mortality.^[2,3]^ More recently, the American Heart Association introduced the concept of cardiovascular–kidney–metabolic (CKM) syndrome to reflect the interconnected relationships among metabolic risk factors, chronic kidney disease, cardiovascular disease, and a continuum of risk.^[4,5]^ Within this contemporary framework, MetS remains an important and operationally defined metabolic risk phenotype and is included among the metabolic risk factors characterizing stage 2 CKM syndrome.^[4]^ Thus, although the CKM framework provides a broader perspective on multisystem cardiometabolic health, MetS continues to serve as a clinically relevant framework for identifying individuals with clustered metabolic abnormalities and elevated long-term cardiometabolic risk.^[2,3]^ However, in clinical practice and population screening, risk stratification often relies on conventional obesity phenotypes or isolated metabolic indicators, which may not adequately capture individuals with concealed high-risk profiles. This underscores the value of simpler and more comprehensive strategies for risk assessment.^[6]^

Insulin resistance and chronic low-grade inflammation are generally considered 2 major biological dimensions of MetS.^[7,8]^ Insulin resistance is closely linked to disturbances in glucose and lipid metabolism and may represent a shared pathway underlying obesity, hypertension, dysglycemia, and atherosclerotic risk.^[7,9]^ The estimated glucose disposal rate (eGDR), which is calculated from routinely available clinical variables, has increasingly been used as a convenient surrogate measure of insulin resistance.^[10,11]^ High-sensitivity C-reactive protein (hs-CRP) is a sensitive circulating marker of systemic inflammation, even at relatively low concentrations, and is commonly used to evaluate inflammatory burden and cardiometabolic risk.^[8]^ Taken together, assessing MetS risk from the interrelated yet non-overlapping perspectives of insulin resistance and inflammation is conceptually well supported.

Although the relationships of eGDR and inflammatory biomarkers with cardiometabolic disorders are increasingly recognized, important questions remain regarding their joint relevance to MetS. Previous studies have shown that lower eGDR is associated with greater inflammatory burden and adverse cardiovascular outcomes.^[12,13]^ Other studies have examined hs-CRP-based markers in relation to cardiovascular disease and glycemic dysregulation,^[14,15]^ as well as the joint associations of eGDR and inflammatory indices with cardiometabolic multimorbidity and mortality.^[16,17]^ In Korea, population-based research has described the prevalence of MetS and its related complications.^[18]^ However, these studies addressed outcomes or research questions that differ from those of the present study. Specifically, evidence remains limited regarding whether eGDR and hs-CRP jointly characterize MetS in a nationally representative general adult population in Korea. Moreover, the dose–response relationships of both markers with MetS, the distribution of risk across joint exposure profiles, and potential heterogeneity across age, body mass index (BMI), and diabetes subgroups have not been comprehensively characterized in this population. Therefore, the purpose of the present study was not to reestablish the known relationship between insulin resistance and inflammation, but to determine whether their combined pattern provides additional phenotypic information regarding MetS in Korean adults using nationally representative KNHANES data.

Based on data from KNHANES 2015–2018, we assessed whether eGDR and hs-CRP were independently related to MetS. We also examined whether these markers showed dose-dependent associations with MetS. Interaction and subgroup analyses were performed to assess whether these associations were consistent across population subgroups and to identify potential heterogeneity. We hypothesized that lower eGDR and higher hs-CRP would each be associated with a higher odds of MetS, and that their combined profiles may correspond to even greater odds.

## 2. Materials and methods

### 2.1. Study design and population

We performed a cross-sectional analysis using data from KNHANES 2015–2018. KNHANES is a nationwide survey administered by the Korea Disease Control and Prevention Agency under a stratified multistage sampling framework with cluster selection. The survey provides nationally representative data on the health and nutritional profiles of the Korean population. The 2015–2017 KNHANES surveys were conducted without separate Institutional Review Board review under the applicable provisions of the Korean Bioethics and Safety Act, whereas the 2018 KNHANES was approved by the Institutional Review Board of the Korea Disease Control and Prevention Agency (IRB No. 2018-01-03-P-A).^[19]^ The present secondary analysis was also reviewed and approved by the Institutional Review Board of Ningde Municipal Hospital (IRB No. 2025-115). Adults aged 19 years or older were eligible for inclusion. To preserve consistency in variable definitions and ensure reproducibility in weighted analyses, we adopted a complete-case strategy. Participants were excluded if information was missing for MetS components, variables required to derive eGDR, HbA1c, hypertension status, hs-CRP, survey design elements (sampling weights, strata, or primary sampling units), or covariates. The final analytical sample consisted of 20,781 participants. The participant flow is presented in Figure 1. In all analyses, the complex sampling design was taken into account by applying the recommended survey weights for the pooled 2015 to 2018 KNHANES dataset.

**Figure 1.**
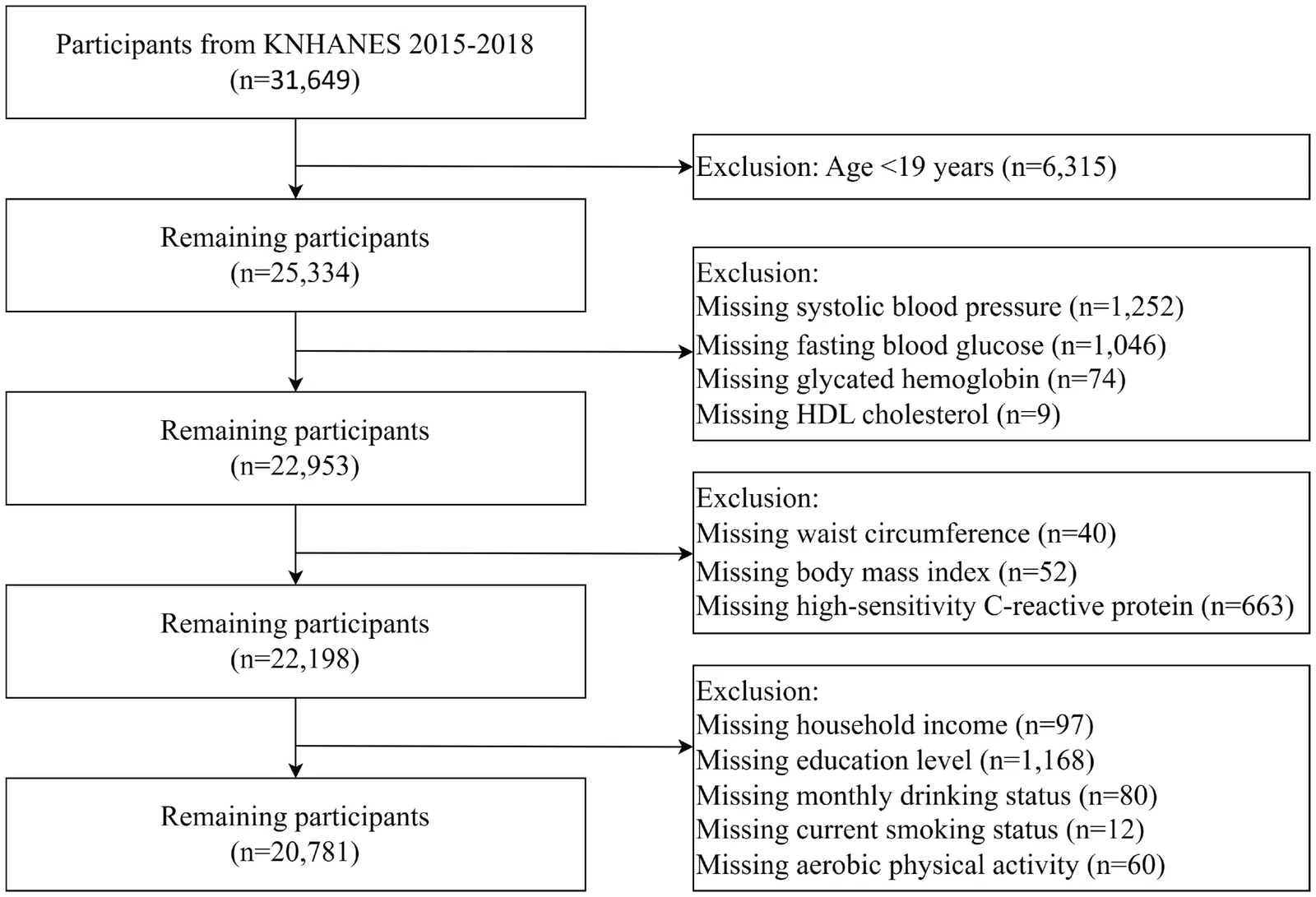
Flowchart of participant selection from KNHANES 2015–2018. HDL = high-density lipoprotein, KNHANES = Korea National Health and Nutrition Examination Survey.

### 2.2. Definition of metabolic syndrome

The definition of MetS was based on the National Cholesterol Education Program Adult Treatment Panel III (NCEP ATP III) framework, with Korean/Asian-specific waist circumference thresholds. Participants with ≥3 of the following 5 criteria were identified as having MetS^[20]^: central obesity, defined as a waist circumference ≥90 cm in men or ≥85 cm in women; elevated blood pressure, defined as systolic blood pressure ≥130 mm Hg and/or diastolic blood pressure ≥85 mm Hg; elevated fasting glucose, defined as ≥100 mg/dL; elevated triglyceride levels, defined as ≥150 mg/dL; and reduced high-density lipoprotein cholesterol, defined as <40 mg/dL in men or <50 mg/dL in women. All MetS components were ascertained from KNHANES physical examinations, laboratory tests, and questionnaire data. Participants were assigned to the MetS or non-MetS group based on these criteria.

### 2.3. Assessment of exposures

The primary exposures were eGDR and hs-CRP. To estimate insulin resistance, eGDR was calculated using a previously reported formula^[21]^: eGDR = 19.02 − (0.22 × BMI [kg/m^2^]) − (3.26 × hypertension status) − (0.61 × HbA1c [%]). Serum hs-CRP levels were measured using standardized laboratory procedures in the KNHANES. Owing to its right-skewed distribution, hs-CRP was log-transformed for continuous analyses, with a constant of 0.01 added prior to transformation to account for near-zero values. In addition, hs-CRP was categorized into quartiles based on weighted distributions.

For joint classification, eGDR and hs-CRP were grouped as low (Q1–Q2) or high (Q3–Q4) according to quartile-based cutoffs. Four joint exposure categories were constructed: high eGDR + low hs-CRP (reference), high eGDR + high hs-CRP, low eGDR + low hs-CRP, and low eGDR + high hs-CRP. A separate binary joint indicator was used in the interaction and subgroup analyses, with the low-eGDR/high-hs-CRP pattern treated as the high-risk group and the high-eGDR/low-hs-CRP pattern as the reference.

Given that eGDR incorporates BMI, HTN status, and HbA1c, which overlap with components of MetS, it was considered a composite metabolic marker rather than an etiologically independent exposure. Accordingly, the findings were interpreted in terms of epidemiological associations rather than causal relationships.

### 2.4. Covariates

Based on the prior literature and potential confounding factors, demographic, socioeconomic, and lifestyle factors were prespecified as covariates. Demographic variables included age (continuous) and sex (male/female). Socioeconomic indicators comprised region, household income quartile, and educational attainment. The lifestyle domain covered current smoking status, drinking in the preceding month, and attainment of recommended aerobic physical activity, each coded as yes/no. These variables were obtained from the KNHANES questionnaires and examination data.

Hypertension, defined as systolic blood pressure ≥130 mm Hg or diastolic blood pressure ≥85 mm Hg, and diabetes, defined as fasting glucose ≥126 mg/dL or HbA1c ≥6.5%, were used for the subgroup analyses. Body mass index, hypertension status, and diabetes were excluded from the primary multivariate models to reduce overadjustment, given their conceptual overlap with elements of the exposures or the outcome.

### 2.5. Statistical analysis

All analyses were performed using R (version 4.4; R Foundation for Statistical Computing). All models incorporated sampling weights, strata (KSTRATA), and primary sampling units (PSU) to reflect the complex survey design. Continuous data were summarized as weighted means with standard errors (SE), whereas categorical data were summarized as weighted percentages. Between-group comparisons were performed using survey-weighted linear regression for continuous variables and Rao–Scott χ^2^ tests for categorical variables.

Associations of eGDR and hs-CRP with MetS were estimated using survey-weighted logistic regression. Three models with increasing levels of adjustment were fitted: an unadjusted model, a model adjusted for age and sex, and a fully adjusted model that additionally included region, income, education, smoking status, alcohol consumption, and physical activity. Results are reported as odds ratios (ORs) with 95% confidence intervals (CIs).

Potential dose–response patterns linking eGDR, log-transformed hs-CRP, and MetS were explored using restricted cubic splines (RCS). Knots were placed at predefined percentiles, with the median used as the reference value. Both overall and nonlinear associations were assessed.

For the joint analyses, joint exposure categories were included in weighted logistic regression models, with the group characterized by high eGDR and low hs-CRP serving as the reference. To test for multiplicative interaction, a cross-product term was added to the fully adjusted model, with hs-CRP entered as a binary variable (high vs low). Predicted probabilities were estimated and visualized to illustrate potential interaction patterns.

We also conducted prespecified subgroup analyses across categories of age, sex, body mass index (BMI), hypertension, and diabetes to examine the stability of the observed associations. Effect modification was evaluated using interaction terms. Estimates were not reported when model convergence was not achieved or when sample sizes were insufficient for stable estimation. A 2-sided *P* value < 0.05 was used to indicate statistical significance.

## 3. Results

### 3.1. Participant characteristics

The participant selection process is shown in Figure 1. A total of 31,649 participants were initially included in the KNHANES 2015–2018. After excluding individuals aged < 19 years (n = 6315) and those with missing key variables, 20,781 adults were included in the final analysis. The weighted prevalence of MetS was 29.4%. The baseline characteristics of the study population are presented in Table 1.

**Table 1:** Weighted baseline characteristics of study participants by metabolic syndrome status.

Variable	Overall	MetS_No	MetS_Yes	*P*_value
	N = 14,41,18,425	N = 10,17,70,723	N = 4,23,47,702	
Age (yr)	46.76 ± 16.22	43.57 ± 15.76	54.42 ± 14.69	<.001
Sex, n (%)				<.001
Male	7,24,95,371 (50.3%)	5,00,87,291 (49.2%)	2,24,08,081 (52.9%)	
Female	7,16,23,053 (49.7%)	5,16,83,432 (50.8%)	1,99,39,621 (47.1%)	
Region, n (%)				.013
Capital area	6,63,90,794 (46.1%)	4,71,59,376 (46.3%)	1,92,31,418 (45.4%)	
Chungcheong	2,40,04,417 (16.7%)	1,67,65,598 (16.5%)	72,38,819 (17.1%)	
Honam	1,42,84,528 (9.9%)	96,37,993 (9.5%)	46,46,535 (11.0%)	
Yeongnam	3,43,91,152 (23.9%)	2,48,38,058 (24.4%)	95,53,094 (22.6%)	
Jeju	9,69,416 (0.7%)	6,64,307 (0.7%)	3,05,109 (0.7%)	
Gangwon	40,78,118 (2.8%)	27,05,390 (2.7%)	13,72,728 (3.2%)	
Income level, n (%)				<.001
Low	3,58,99,348 (24.9%)	2,41,00,811 (23.7%)	1,17,98,537 (27.9%)	
Lower-middle	3,60,29,327 (25.0%)	2,52,53,671 (24.8%)	1,07,75,656 (25.4%)	
Upper-middle	3,61,39,347 (25.1%)	2,57,82,500 (25.3%)	1,03,56,847 (24.5%)	
High	3,60,50,402 (25.0%)	2,66,33,740 (26.2%)	94,16,662 (22.2%)	
Education level, n (%)				<.001
Elementary school or below	2,08,26,238 (14.5%)	98,99,268 (9.7%)	1,09,26,971 (25.8%)	
Middle school	1,25,81,604 (8.7%)	74,59,118 (7.3%)	51,22,486 (12.1%)	
High school	5,18,09,285 (35.9%)	3,85,24,815 (37.9%)	1,32,84,470 (31.4%)	
College or above	5,89,01,298 (40.9%)	4,58,87,521 (45.1%)	1,30,13,776 (30.7%)	
Smoking status, n (%)				<.001
No	11,31,41,106 (78.5%)	8,11,28,928 (79.7%)	3,20,12,178 (75.6%)	
Yes	3,09,77,319 (21.5%)	2,06,41,794 (20.3%)	1,03,35,524 (24.4%)	
Alcohol consumption, n (%)				<.001
No	5,83,83,647 (40.5%)	3,94,32,188 (38.7%)	1,89,51,459 (44.8%)	
Yes	8,57,34,778 (59.5%)	6,23,38,535 (61.3%)	2,33,96,243 (55.2%)	
Aerobic physical activity				<.001
No	7,52,04,483 (52.2%)	4,98,73,620 (49.0%)	2,53,30,863 (59.8%)	
Yes	6,89,13,942 (47.8%)	5,18,97,103 (51.0%)	1,70,16,839 (40.2%)	
Hypertension, n (%)				<.001
No	10,27,28,169 (71.3%)	8,53,80,467 (83.9%)	1,73,47,701 (41.0%)	
Yes	4,13,90,256 (28.7%)	1,63,90,255 (16.1%)	2,50,00,001 (59.0%)	
Diabetes, n (%)				<.001
No	12,97,41,473 (90.0%)	9,74,12,881 (95.7%)	3,23,28,592 (76.3%)	
Yes	1,43,76,952 (10.0%)	4357,841 (4.3%)	1,00,19,110 (23.7%)	
eGDR	9.37 ± 1.98	10.11 ± 1.49	7.57 ± 1.87	<.001
eGDR quartiles, n (%)				<.001
Q1	3,30,80,694 (23.0%)	94,65,639 (9.3%)	2,36,15,055 (55.8%)	
Q2	3,42,24,350 (23.7%)	2,09,96,174 (20.6%)	1,32,28,175 (31.2%)	
Q3	3,77,55,827 (26.2%)	3,28,40,919 (32.3%)	49,14,908 (11.6%)	
Q4	3,90,57,555 (27.1%)	3,84,67,991 (37.8%)	5,89,564 (1.4%)	
hsCRP (mg/L)	1.17 ± 1.97	1.01 ± 1.86	1.56 ± 2.18	<.001
hs-CRP quartiles, n (%)				<.001
Q1	3,72,46,746 (25.8%)	3,25,70,936 (32.0%)	46,75,811 (11.0%)	
Q2	3,64,30,193 (25.3%)	2,77,68,882 (27.3%)	86,61,310 (20.5%)	
Q3	3,58,04,823 (24.8%)	2,25,84,021 (22.2%)	1,32,20,801 (31.2%)	
Q4	3,46,36,663 (24.0%)	1,88,46,883 (18.5%)	1,57,89,780 (37.3%)	
BMI (kg/m^2^)	23.99 ± 3.59	22.95 ± 3.09	26.49 ± 3.48	<.001
Waist circumference (cm)	82.48 ± 10.26	79.20 ± 9.04	90.34 ± 8.62	<.001
Fasting glucose, mg/dL	99.98 ± 23.72	94.70 ± 16.83	112.67 ± 31.72	<.001
HbA1c, %	5.64 ± 0.79	5.46 ± 0.59	6.07 ± 1.01	<.001
Total cholesterol (mg/dL)	192.64 ± 36.68	189.82 ± 34.27	199.44 ± 41.12	<.001
HDL-C (mg/dL)	51.10 ± 12.62	54.43 ± 12.11	43.10 ± 9.95	<.001
LDL-C (mg/dL)	115.80 ± 32.48	113.45 ± 30.49	118.77 ± 34.63	<.001
Triglycerides (mg/dL)	139.30 ± 120.75	106.00 ± 71.27	219.33 ± 168.37	<.001
eGFR (mL/min/1.73m^2^)	101.05 ± 16.62	103.62 ± 15.80	94.88 ± 16.92	<.001

Continuous variables are presented as weighted means (standard errors), and categorical variables as weighted percentages.

BMI = body mass index, eGDR = estimated glucose disposal rate, eGFR = estimated glomerular filtration rate, HDL-C = high-density lipoprotein cholesterol, hs-CRP = high-sensitivity C-reactive protein, LSL-C = low-density lipoprotein cholesterol, MetS = metabolic syndrome.

Participants with MetS tended to be older, included a greater proportion of men, and showed less advantaged socioeconomic characteristics, including lower income and educational attainment, than those without MetS (all *P* < .001). Current smoking was more common, whereas achievement of recommended aerobic physical activity was less common among participants with MetS (all *P* < .001). Regarding key metabolic and inflammatory indicators, the MetS group showed lower eGDR and higher hs-CRP levels (both *P* < .001). In addition, BMI, waist circumference, fasting glucose, HbA1c, total cholesterol, low-density lipoprotein cholesterol, and triglyceride levels were higher in the MetS group, whereas high-density lipoprotein cholesterol and estimated glomerular filtration rate were lower (all *P* < .001).

### 3.2. Associations of eGDR and hs-CRP with MetS

Table 2 summarizes the findings from the survey-weighted logistic regression analyses. eGDR was inversely associated with MetS. After full adjustment, a 1-unit increment in eGDR corresponded to reduced odds of MetS (OR 0.48, 95% CI: 0.47–0.49, *P* < .001). A graded inverse association was observed across quartiles, with progressively lower odds of MetS at higher eGDR levels (*P* for trend < .001).

**Table 2:** Associations of eGDR and hs-CRP with metabolic syndrome.

Variable	Model 1 OR (95% CI)	Model 2 OR (95% CI)	Model 3 OR (95% CI)
eGDR (per 1-unit increase)	0.471 (0.462, 0.481)	0.484 (0.473, 0.494),	0.481 (0.470, 0.492)
eGDR quartiles			
Q1 (reference)	Reference	Reference	Reference
Q2	0.253 (0.229, 0.279)	0.246 (0.222, 0.272)	0.237 (0.213, 0.263)
Q3	0.060 (0.053, 0.067)	0.063 (0.056, 0.071)	0.062 (0.055, 0.070)
Q4	0.006 (0.005, 0.008)	0.007 (0.006, 0.009)	0.007 (0.005, 0.009)
*P* for trend	*P* < .001	*P* < .001	*P* < .001
log hs-CRP (per 1-unit increase)	1.789 (1.720, 1.862)	1.739 (1.666, 1.815)	1.714 (1.642, 1.790)
hsCRP quartiles			
Q1 (reference)	Reference	Reference	Reference
Q2	2.173 (1.929, 2.447)	1.937 (1.720, 2.180)	1.927 (1.712, 2.170)
Q3	4.078 (3.641, 4.567)	3.541 (3.159, 3.969)	3.464 (3.090, 3.883)
Q4	5.836 (5.209, 6.538)	5.211 (4.641, 5.851)	5.044 (4.489, 5.669)
*P* for trend	*P* < .001	*P* < .001	*P* < .001

Model 1: unadjusted.

Model 2: adjusted for age and sex.

Model 3: fully adjusted for age, sex, region, income, education, smoking status, alcohol consumption, and physical activity.

Odds ratios (ORs) and 95% confidence intervals (CIs) were estimated using survey-weighted logistic regression models. eGDR and hs-CRP were analyzed as continuous variables and quartiles, with *P* for trend calculated across quartiles.

CI = confidence interval, eGDR = estimated glucose disposal rate, hs-CRP = high-sensitivity C-reactive protein, OR = odds ratio.

In contrast, hs-CRP was positively associated with MetS. Model 3 showed that higher log-transformed hs-CRP corresponded to greater odds of MetS (OR 1.71, 95% CI: 1.64–1.79, *P* < .001). A graded positive association was observed across quartiles, with progressively higher odds of MetS at increasing hs-CRP levels (*P* for trend < .001).

### 3.3. Dose–response relationships

Dose–response patterns linking eGDR, hs-CRP, and MetS were further examined using restricted cubic splines (Fig. 2). The spline for eGDR indicated a steadily decreasing pattern in the odds of MetS, with some flattening at the upper end of the distribution. In contrast, log-transformed hs-CRP exhibited a positive association with MetS, with a more pronounced increase in odds at moderate-to-high levels and a tendency toward attenuation at the upper range.

**Figure 2.**
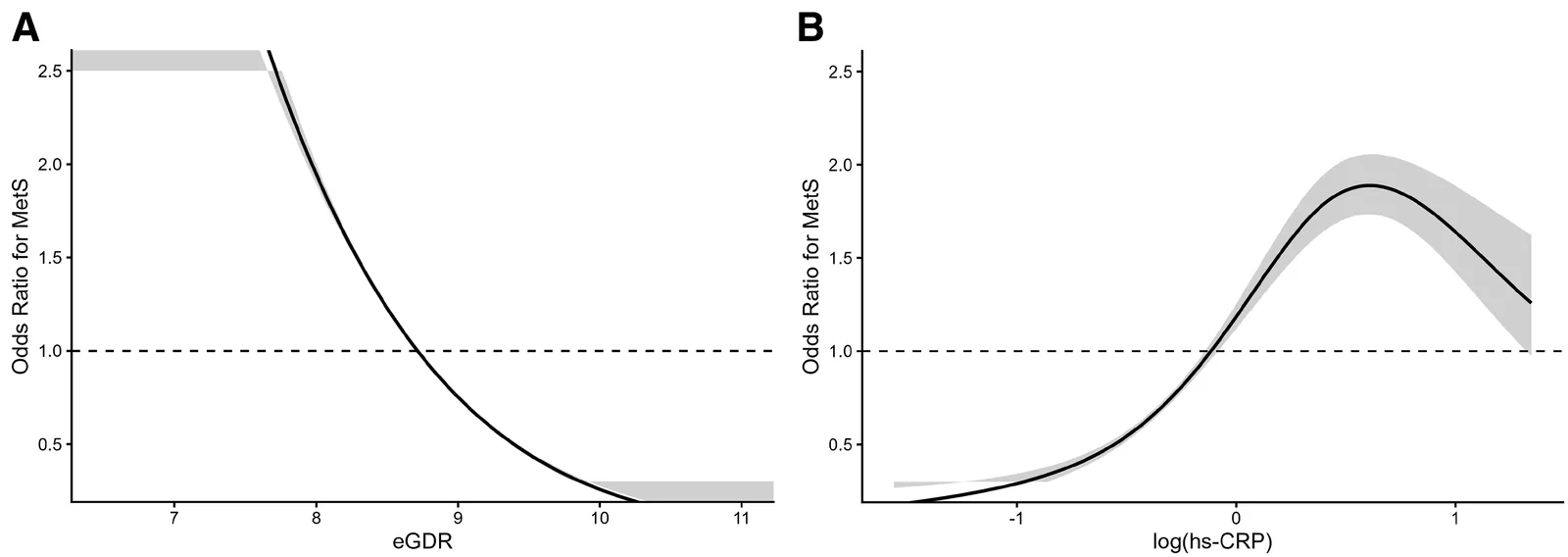
Dose–response relationships of eGDR and hs-CRP with metabolic syndrome. Adjusted odds ratios (ORs) and 95% confidence intervals (CIs) were estimated using restricted cubic spline models. (A) eGDR; (B) log-transformed hs-CRP. The horizontal dashed line indicates OR = 1.0. CI = confidence interval, eGDR = estimated glucose disposal rate, hs-CRP = high-sensitivity C-reactive protein, MetS = metabolic syndrome, OR = odds ratio.

### 3.4. Joint associations of eGDR and hs-CRP with MetS

The joint association results are presented in Table 3. With the high-eGDR/low-hs-CRP pattern as the reference category, a stepwise increase in the odds of MetS was observed across the other joint exposure groups after full adjustment. A clear gradient was evident, with the combination of low eGDR and high hs-CRP associated with substantially higher odds of MetS.

**Table 3:** Joint association of eGDR and hs-CRP with metabolic syndrome.

Variable	Model 1 OR (95% CI)	Model 2 OR (95% CI)	Model 3 OR (95% CI)
High eGDR + Low hsCRP	Reference	Reference	Reference
High eGDR + High hsCRP	2.744 (2.328, 3.234)	2.501 (2.118, 2.953)	2.413 (2.045, 2.849)
Low eGDR + Low hsCRP	16.374 (14.157, 18.938)	12.581 (10.836, 14.607)	12.452 (10.721, 14.461)
Low eGDR + High hsCRP	33.190 (29.034, 37.941)	27.762 (24.171, 31.886)	27.132 (23.597, 31.198)
*P* for interaction	*P* < .001	*P* < .001	*P* < .001

Model 1: unadjusted.

Model 2: adjusted for age and sex.

Model 3: fully adjusted for age, sex, region, income, education, smoking status, alcohol consumption, and physical activity.

Odds ratios (ORs) and 95% confidence intervals (CIs) were estimated across joint exposure categories using survey-weighted logistic regression models.

CI = confidence interval, eGDR = estimated glucose disposal rate, hs-CRP = high-sensitivity C-reactive protein, OR = odds ratio.

### 3.5. Interaction analysis

A statistically significant multiplicative interaction between eGDR and hs-CRP was not detected (*P* for interaction = .064; Fig. 3). The predicted probability curves indicated that, across the range of eGDR, the probability of MetS remained consistently higher in the high hs-CRP group than in the low hs-CRP group. In both groups, the probability of MetS decreased with increasing eGDR, with no apparent crossing of the curves.

**Figure 3.**
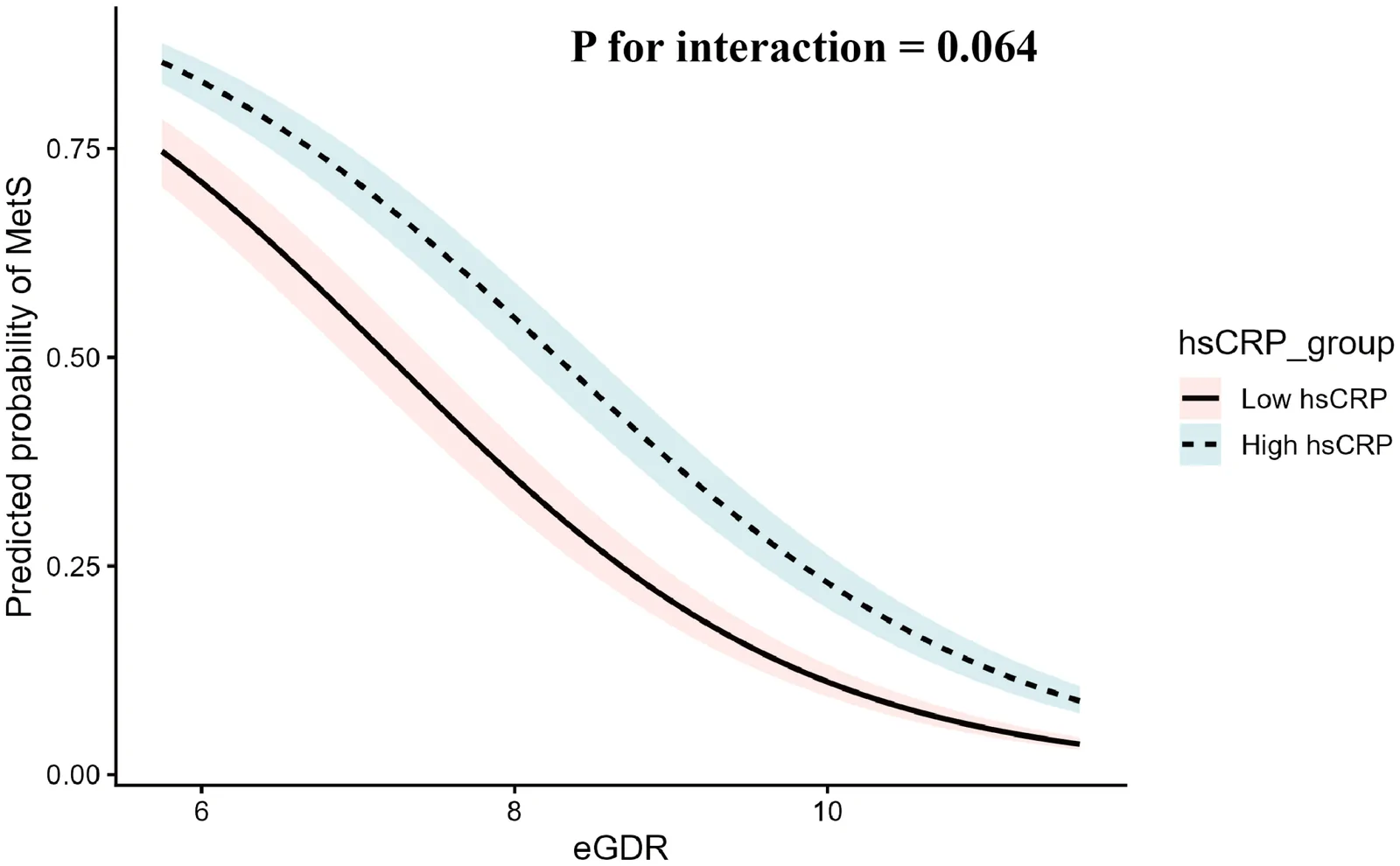
Interaction between eGDR and hs-CRP in relation to metabolic syndrome risk. Adjusted predicted probabilities of metabolic syndrome (MetS) across eGDR levels, stratified by hs-CRP groups. Solid and dashed lines represent low and high hs-CRP groups, respectively, with shaded areas indicating 95% confidence intervals (CIs). The *P* value for multiplicative interaction was .064. CI = confidence interval, eGDR = estimated glucose disposal rate, hs-CRP = high-sensitivity C-reactive protein, MetS = metabolic syndrome.

### 3.6. Subgroup analyses

The subgroup analysis results are presented in Table 4. Overall, the association between the joint high-risk group (low eGDR and high hs-CRP) and MetS remained statistically significant across most subgroups. The association appeared stronger among individuals aged <60 years than among those aged ≥60 years, with evidence of effect modification (*P* for interaction < .001). No significant interaction was observed according to sex (*P* for interaction = .976). Although the association appeared more pronounced in individuals with BMI < 25 kg/m^2^, no statistically significant interaction was detected (*P* for interaction = .057). A significant interaction was observed according to diabetes status, with a stronger association among individuals without diabetes (*P* for interaction = .024). Estimates were not reported for hypertension-stratified analyses because of the insufficient sample size in the reference group.

**Table 4:** Subgroup analyses of the joint association between eGDR and hs-CRP with metabolic syndrome.

Subgroup	OR (95% CI)	*P*	*P* for interaction
Age			<.001
<60	4.690 (3.506, 6.274)	<.001	
≥60	2.055 (1.528, 2.764)	<.001	
Sex			.976
Male	3.062 (2.195, 4.272)	<.001	
Female	3.180 (2.350, 4.310)	<.001	
BMI, kg/m^2^			.057
<25	7.480 (4.998, 11.193)	<.001	
≥25	4.624 (3.276, 6.528)	<.001	
Hypertension			—
Yes	4.909 (3.733, 6.455)	<.001	
No	Not estimable^*^	—	
Diabetes	3.367 (2.619, 4.329)	<.001	.024
No	2.05 (1.40, 3.01)	.001	
Yes	4.909 (3.733, 6.455)	<.001	

Model: fully adjusted for age, sex, region, income, education, smoking status, alcohol consumption, and physical activity.

Adjusted odds ratios (ORs) and 95% confidence intervals (CIs) were estimated across predefined subgroups, with interaction terms used to assess effect modification.

CI = confidence interval, eGDR = estimated glucose disposal rate, hs-CRP = high-sensitivity C-reactive protein, OR = odds ratio.

* Not estimable: The adjusted odds ratio and its 95% CI could not be estimated for this subgroup.

## 4. Discussion

In Korean adults from a nationally representative dataset, we assessed the separate and combined relationships of eGDR and hs-CRP with MetS. Lower eGDR and higher hs-CRP were independently associated with MetS, and both showed nonlinear dose–response patterns. Among the combined exposure profiles, the low-eGDR/high-hs-CRP pattern was associated with the greatest odds of MetS. The observed associations were stronger in younger participants, those with BMI < 25 kg/m^2^, and those without diabetes. Taken together, these findings suggest that integrating information on insulin resistance and inflammation may contribute to identifying adverse metabolic profiles beyond BMI-defined risk and may provide additional context for metabolic risk characterization within the broader cardiovascular-kidney-metabolic (CKM) framework.

Our findings should be interpreted as an extension of, rather than a replication of, the existing evidence linking insulin resistance and inflammation. Previous studies have demonstrated associations between lower eGDR, systemic inflammation, and adverse cardiovascular outcomes.^[12,13]^ In contrast, the present study addressed a different question by examining MetS as the outcome in a nationally representative sample of Korean adults across a broad adult age range. Our contribution lies in characterizing this relationship at several complementary levels, including continuous and quartile-based associations, nonlinear dose–response patterns, joint eGDR/hs-CRP profiles, and heterogeneity across clinically relevant population subgroups. The observation that the low-eGDR/high-hs-CRP profile was associated with the greatest odds of MetS, together with heterogeneity according to age and diabetes status, further characterizes the population distribution of this combined metabolic-inflammatory phenotype. These findings do not establish a new causal relationship or demonstrate improved prediction; rather, they refine the population-level characterization of the combined metabolic and inflammatory phenotype in relation to MetS. Previous population-based studies have reported associations between lower eGDR and MetS.^[22,23]^ Similarly, epidemiological studies have linked elevated C-reactive protein levels to MetS and its development.^[24-26]^ These findings are consistent with previous evidence highlighting the interconnected roles of insulin resistance and inflammation in metabolic dysfunction.^[27]^

These findings are biologically plausible. eGDR integrates BMI, blood pressure, and HbA1c and may reflect overall insulin sensitivity and metabolic burden rather than a single metabolic component. Insulin resistance is widely regarded as a central feature of MetS and is linked to multiple metabolic abnormalities.^[28-30]^ Similarly, hs-CRP reflects systemic low-grade inflammation and has been associated with metabolic dysregulation.^[28-31]^ It may capture upstream inflammatory processes related to adipose tissue dysfunction and immune activation. Taken together, the combination of low eGDR and high hs-CRP may reflect a coexisting state of metabolic imbalance and inflammatory activation rather than 2 independent signals.

The stronger joint association observed in categorical analyses, alongside the lack of statistically significant multiplicative interaction, is not contradictory. Differences in findings may arise from the analytical framework, as interaction tests are scale-dependent and often more conservative.^[32,33]^ Categorical joint analyses emphasize contrasts between extreme exposure groups, whereas multiplicative interaction models assess changes in slope. Consistent with this, the predicted probability curves showed parallel patterns without a clear crossing. This suggests that hs-CRP may shift overall risk levels rather than substantially modifying the association between eGDR and MetS.

The association remained consistent across most subgroups, although some heterogeneity in magnitude was observed. Notably, the stronger association among younger adults in the present study differs from findings of a recent prospective Chinese study of incident cardiovascular disease, in which the joint association of eGDR and hs-CRP was more pronounced among older individuals.^[13]^ This difference may reflect differences in study populations, outcome definitions, and study design: our analysis focused on prevalent MetS across a broad adult age range, whereas the previous study evaluated incident cardiovascular disease in a middle-aged and older population. In contrast, the stronger association observed among individuals without diabetes in our study is broadly consistent with the pattern reported in that prospective study.^[13]^ These findings suggest that age and diabetes status may influence the population distribution of the combined metabolic-inflammatory phenotype, although the underlying mechanisms cannot be determined from the present cross-sectional analysis. Age-related changes in chronic low-grade inflammation and metabolic regulation may provide a plausible context for the observed heterogeneity,^[34,35]^ although this explanation remains speculative. The more pronounced association in individuals with a BMI < 25 kg/m^2^ is consistent with the concept of metabolically unhealthy normal weight.^[36–38]^ No clear effect modification by sex was observed, and the findings for hypertension should be interpreted with caution because of the limited sample size.

These results may have practical implications for both clinical care and public health. MetS reflects the clustering of multiple metabolic abnormalities and represents an important metabolic risk phenotype within the broader cardiovascular-kidney-metabolic (CKM) framework.^[4,5]^ Insulin resistance and chronic low-grade inflammation are closely interconnected biological processes that may contribute to the continuum linking metabolic dysfunction with cardiovascular and kidney disease. In the present study, the combination of lower eGDR and higher hs-CRP identified individuals with a particularly unfavorable metabolic profile, and this association remained evident across most population subgroups. Notably, the association appeared stronger among younger individuals and those without diabetes, suggesting that the joint assessment of eGDR and hs-CRP may provide complementary information for characterizing metabolic risk before overt cardiometabolic disease becomes established. Considering eGDR and hs-CRP together may therefore complement the characterization of adverse metabolic profiles that are not fully captured by BMI-defined risk alone and may offer a pragmatic approach to metabolic risk characterization within the broader CKM framework. However, these markers should not be interpreted as a substitute for formal CKM staging, and prospective studies are required to determine whether their joint assessment improves prediction of future CKM-related outcomes.

## 5. Strengths and limitations

Several features of this study strengthen the interpretation of the findings. First, reliance on nationally representative KNHANES data improves the population relevance of the results, and all analyses incorporated a complex survey framework. Second, the study comprehensively evaluated the independent associations, dose–response relationships, and joint effects of eGDR and hs-CRP with MetS. Third, interaction and subgroup analyses were conducted to provide a more complete assessment of these associations. Several limitations also warrant attention. First, because of the cross-sectional design, the temporal sequence between the studied variables and MetS could not be determined. Second, eGDR shares components with MetS, and the observed associations may partially reflect shared components. Third, although multiple covariates were adjusted for, residual confounding remains possible, and factors not captured in the dataset, including dietary patterns and medication use, may have affected the findings. Fourth, hs-CRP was based on a single measurement and may be affected by short-term variability or subclinical conditions. Fifth, although eGDR is a practical surrogate, it does not fully capture insulin sensitivity as measured by gold-standard methods. Finally, some subgroup-specific estimates were constrained by small numbers within the reference category; therefore, these findings require cautious interpretation.

## 6. Conclusions

In this nationally representative sample of Korean adults, lower eGDR and higher hs-CRP levels were independently associated with MetS, and the combined low-eGDR/high-hs-CRP profile was associated with the highest odds of MetS. Although no statistically significant multiplicative interaction was observed, the joint association was generally consistent across population subgroups and appeared stronger among younger adults and individuals without diabetes. Rather than establishing a novel biological relationship between insulin resistance and inflammation, these findings extend previous evidence by characterizing their joint relationship with MetS across dose–response patterns and clinically relevant subgroups in a nationally representative Korean population. Further prospective studies are needed to determine whether these cross-sectional patterns translate into improved prediction of future CKM-related outcomes.

## Acknowledgments

The authors thank the Korea Disease Control and Prevention Agency (KDCA) for granting access to the Korea National Health and Nutrition Examination Survey (KNHANES) data.

## Author contributions

**Conceptualization:** Linling Ruan, Shuangyu Ou.

**Data curation:** Linling Ruan.

**Formal analysis:** Linling Ruan.

**Investigation:** Linling Ruan.

**Resources:** Shuangyu Ou.

**Supervision:** Shuangyu Ou.

**Writing – original draft:** Linling Ruan.

**Writing – review & editing:** Shuangyu Ou.
